# Fu’s Subcutaneous Needling treatment for migraine without aura: a randomized controlled trial protocol

**DOI:** 10.3389/fneur.2025.1689562

**Published:** 2026-01-12

**Authors:** Wenqi Guo, Yusha Yang, Jian Sun

**Affiliations:** 1 The Second Clinical College of Guangzhou University of Chinese Medicine, Guangzhou, Guangdong, China; 2 Clinical Medical College of Acupuncture, Moxibustion and Rehabilitation, Guangzhou University of Chinese Medicine, Guangzhou, Guangdong, China

**Keywords:** Fu’s Subcutaneous Needling, migraine without aura, flunarizine hydrochloride, near-infrared spectroscopy, randomized controlled trial

## Abstract

**Introduction:**

Fu’s Subcutaneous Needling (FSN) is a novel acupuncture technique that has demonstrated significant efficacy in pain management. However, its effectiveness in treating migraine without aura (MWoA) has not been systematically validated. Therefore, this study aims to evaluate the analgesic efficacy of FSN and its impact on headache frequency and patients’ quality of life (QoL) in comparison with guideline-recommended pharmacotherapy (flunarizine hydrochloride). Additionally, this study will assess the influence of FSN on hemodynamic changes in the prefrontal cortex to further elucidate its underlying mechanisms.

**Methods:**

This study is a randomized controlled clinical trial involving 44 participants, who are randomly allocated at a 1:1 ratio to either the FSN group or the medication group. Participants in the FSN group receive FSN treatment twice weekly for 4 weeks, while those in the medication group are administered 5 mg oral flunarizine hydrochloride capsules nightly for the same duration. Both groups are followed up for 3 months post-treatment. The primary outcome is the change in monthly migraine days (MMDs). From baseline after 4 weeks of treatment. Secondary outcome measures including the Migraine-Specific Quality of Life (MSQOL) score, headache diary records, Migraine Disability Assessment (MIDAS), Visual Analog Scale (VAS) scores and near-infrared spectroscopy (NIRS) are also used to validate clinical efficacy.

## 1 Introduction

Migraine is a primary headache disorder marked by recurrent episodes of moderate to severe throbbing pain, often accompanied by symptoms such as nausea, vomiting, photophobia, or phonophobia (1). It adversely affects patients’ daily activities and quality of life to varying degrees (2, 3). According to the 2015 Global Burden of Diseases (GBD) study, migraine ranks as the second most prevalent neurological disorder associated with disability (38). Epidemiological surveys indicate that the annual prevalence of migraine in China is 9.3%,with a female-to-male ratio of approximately 2:1 (4). Migraine remains a predominant cause of disability under 50 (5). Currently, the etiology and pathogenesis of migraine remain incompletely understood, and no unified or definitive diagnostic criteria have been established. The primary treatment recommended by clinical guidelines is pharmacotherapy, including preventive medications (e.g., flunarizine hydrochloride) and acute analgesics (e.g., nonsteroidal anti-inflammatory drugs) (1). However, clinical practice often faces challenges such as inadequate preventive treatment and overuse of acute analgesics (6, 7), potentially exacerbating chronic migraine progression or triggering medication-overuse headache (8). Therefore, there is a pressing need to explore safer and more effective non-pharmacological interventions to prevent the onset and progression of migraine.

**Figure 1:**
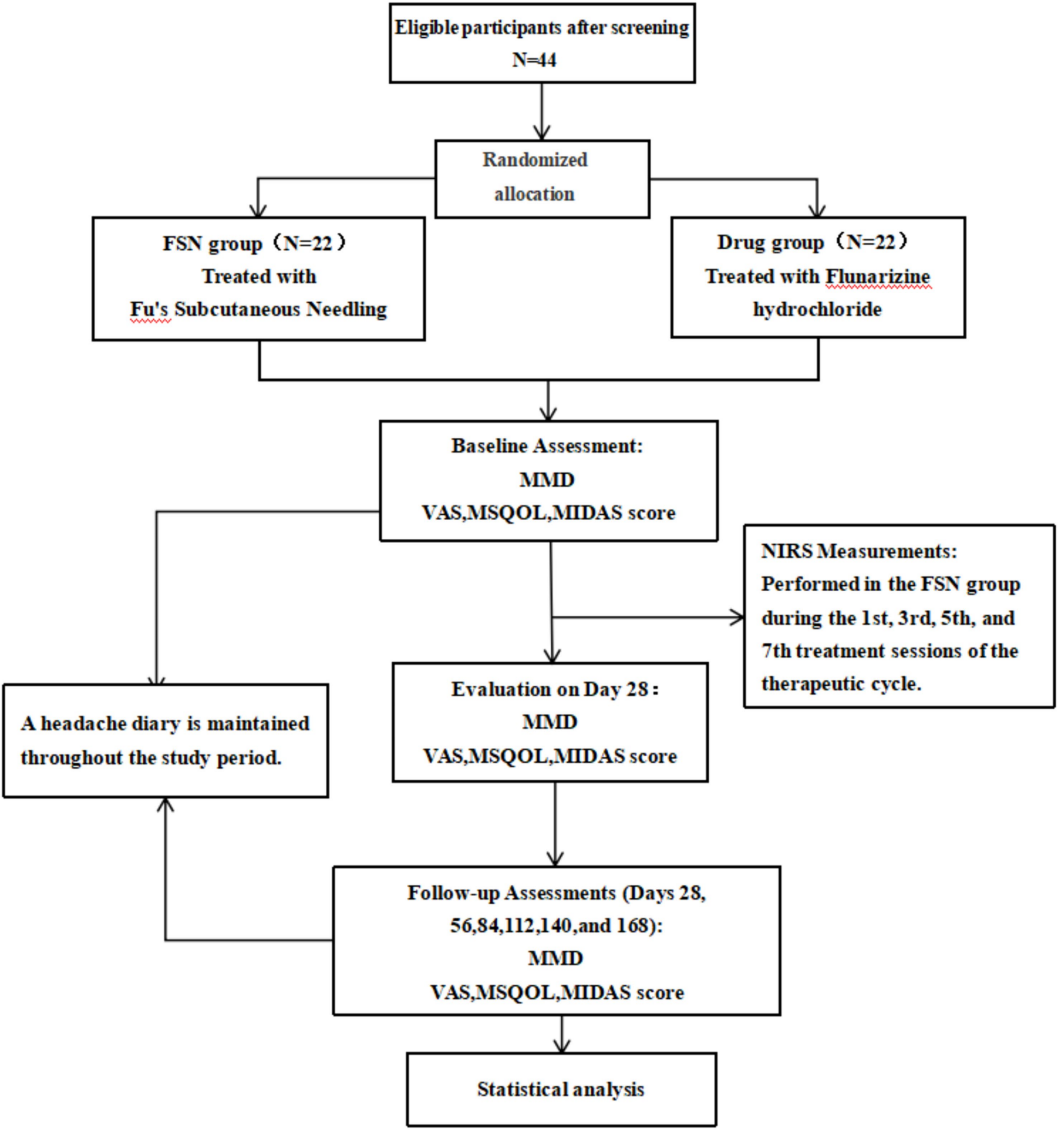
Study protocol flowchart.

**Figure 2:**
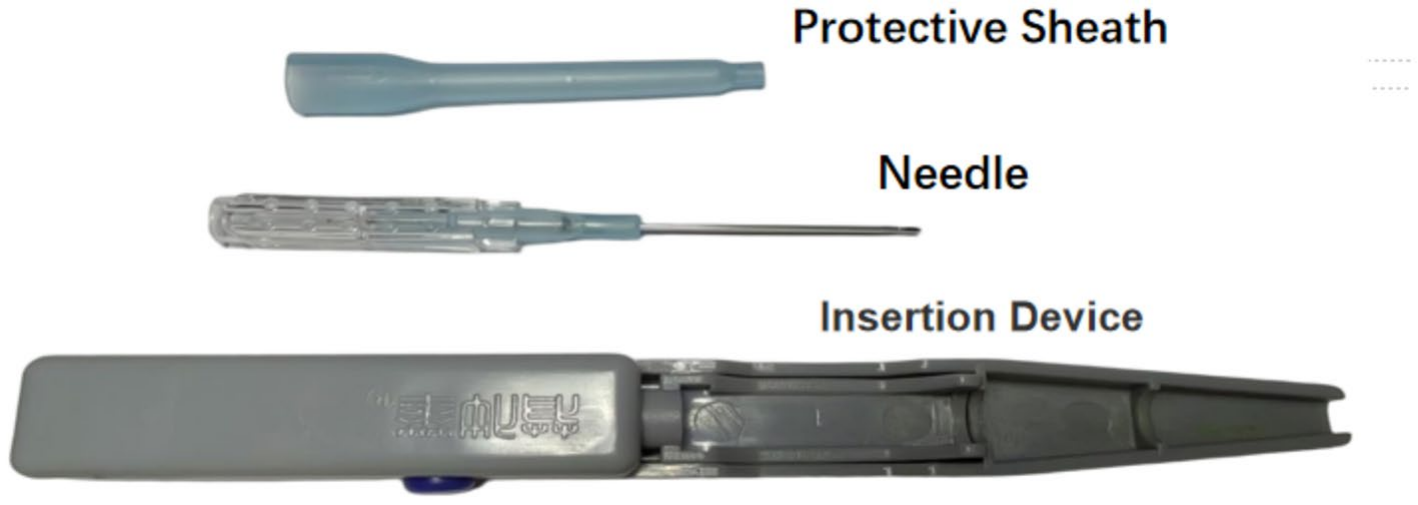
FSN and insertion device.

**Figure 3:**
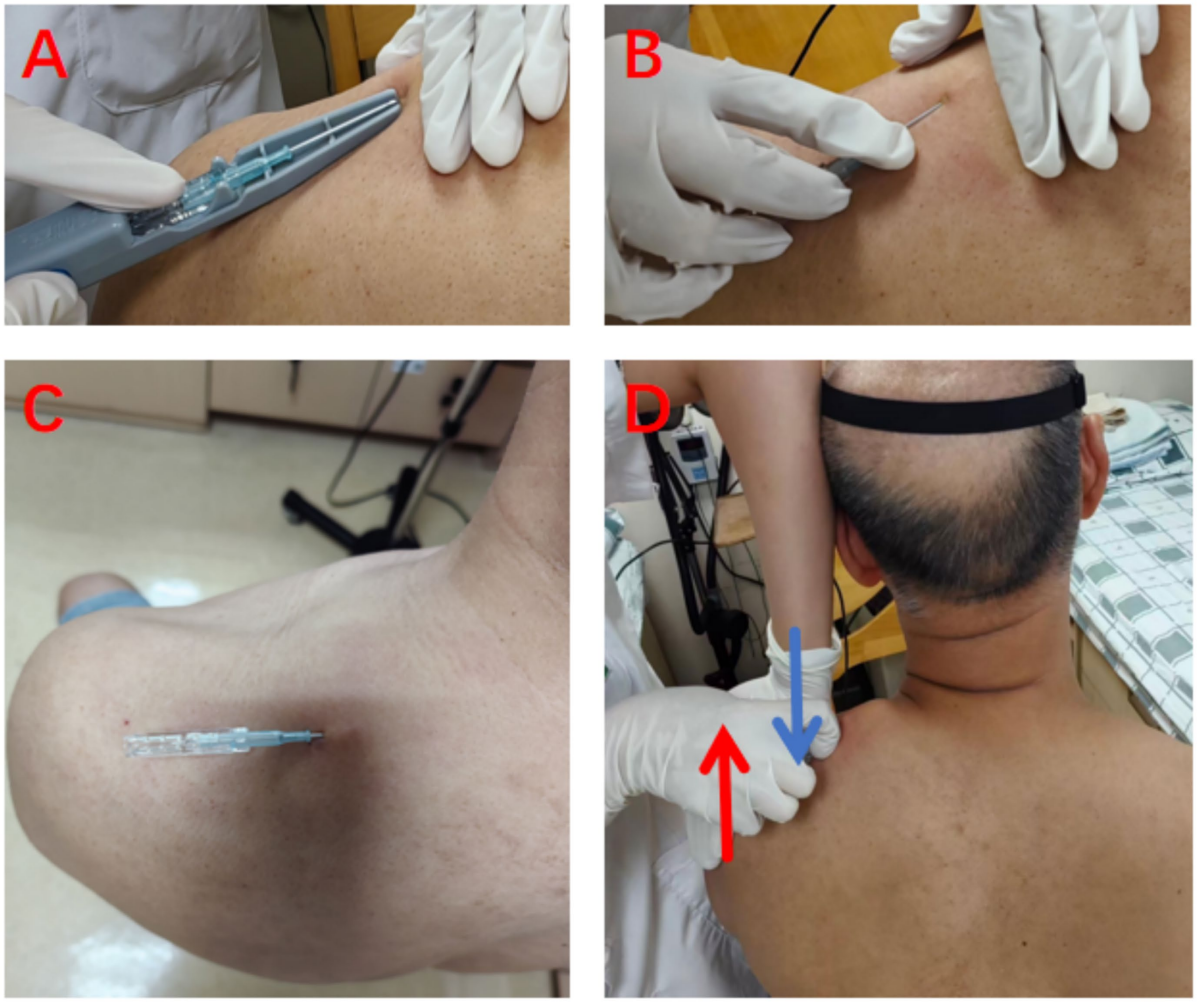
Schematic Illustration of FSN Procedure. Using the Trapezius Muscle as an Example: **A–C**: the insertion device is pressed against the skin, then the needle is inserted subcutaneously parallel to the skin surface. **D**: upon completion of needle insertion, the operator performs lateral sweeping motions with the index and ring fingers of one hand while applying resistance with the other hand against the participant, eliciting a shrugging motion with resistance.

**Figure 4:**
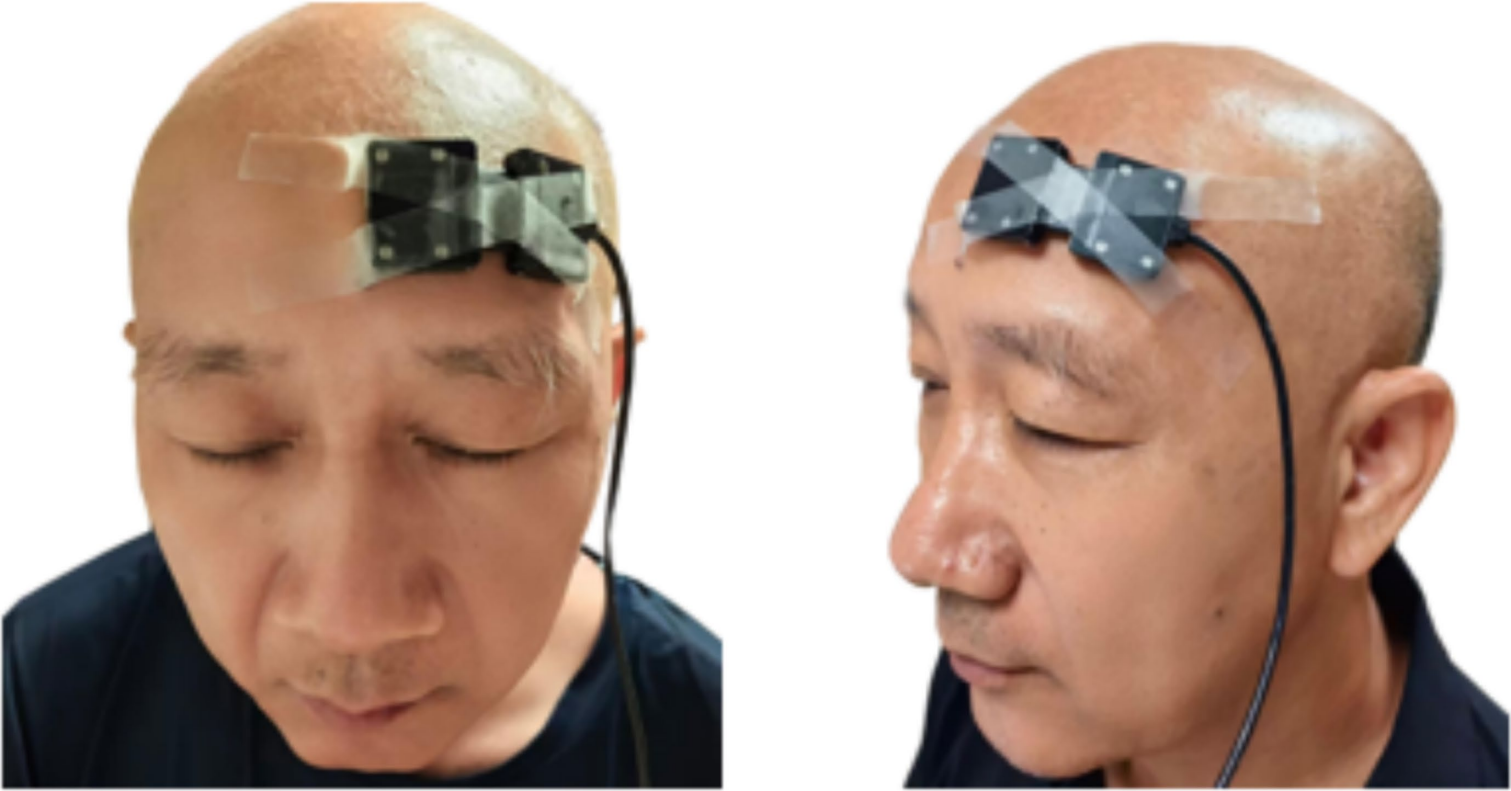
Placement of NIRS.

Emerging evidence indicates that myofascial trigger points (MTrPs) play a significant role in migraine, particularly in migraine without aura (MWoA) (9–11). Compared with non-migraine individuals, MWoA patients exhibit localized tenderness and referred pain in pericranial and cervical muscles that can trigger headaches. The tender points in pericranial muscles are more sensitive even during headache-free periods, and headache intensity correlates positively with tenderness severity. Moreover, the convergence of referred pain from different pericranial muscles can elicit typical unilateral or bilateral migraine-like pain (9–11). The pain sensation elicited by pressing MTrPs in these regions resembles the prodromal symptoms of migraine attacks (12), and the referred pain areas overlap with migraine attack sites (9). Previous studies have applied therapeutic interventions such as acupuncture and repetitive peripheral magnetic stimulation (rPMS) targeting MTrPs in the sternocleidomastoid and trapezius muscles of patients with MWoA. These interventions are found to significantly reduce headache intensity scores and attack frequency post-treatment (13, 14). In summary, MTrPs in migraine patients are predominantly localized in the upper trapezius, sternocleidomastoid, splenius capitis, and suboccipital muscle groups (9, 10, 15–18), which may provide valuable anatomical references for clinical migraine management (see Figures 1–4; Table 1).

**Table 1:** Study schedule.

Stage	Baseline	Treatment			Follow-up (after treatment)		
Time point	Day 0	Day 1	→	Day 28	Day 28	→	Day 168
Enrolment							
Eligibility	×						
Informed consent	×						
Allocation	×						
Interventions							
FSN		×					
Flunarizine hydrochloride		×					
Assessment							
MMD	×			×	×	×	×
MSQOL scores	×			×	×	×	×
VAS scores	×			×	×	×	×
MIDAS scores	×			×	×	×	×
Headache diaries	×			×	×	×	×
NIRS		4 measurements					
Adverse events	×	×					

Fu’s Subcutaneous Needling (FSN), developed by Professor Fu Zhonghua, is a novel acupuncture technique derived from traditional Chinese medicine and grounded in anatomy, physiology, and myofascial trigger point theory. It involves repetitive sweeping of the subcutaneous loose connective tissue over pathologically tense muscles to reduce peripheral muscle tension (19), combined with resistance exercises to promote tissue reperfusion. This approach rapidly alleviates muscle ischemia and hypoxia (20), reduces mechanical compression on surrounding tissues, and demonstrates notable efficacy in pain management (21, 22).

Currently, there is a paucity of clinical studies on the application of floating acupuncture for MWoA, and objective assessments of its therapeutic effects are lacking. Therefore, we propose a randomized controlled trial to assess the clinical efficacy and underlying mechanisms of FSN in treating MWoA, with the goal of identifying safer and more effective treatment strategies. We hypothesize that floating acupuncture is non-inferior to flunarizine hydrochloride in alleviating pain intensity, reducing attack frequency, and enhancing quality of life in MWoA patients.

## 2 Methods and analysis

### 2.1 Study design

This randomized controlled clinical trial will take place at Guangdong Provincial Hospital of Traditional Chinese Medicine between April 2025 and April 2026, following the Declaration of Helsinki guidelines. The hospital’s Ethics Committee approved the study (Approval No.: YF2025-127), which has been registered on a clinical trial platform (NCT07068815).

### 2.2 Study setting

Participants are recruited from the acupuncture outpatient clinic and neurology outpatient clinic of Guangdong Provincial Hospital of Traditional Chinese Medicine. Eligible participants who provide consent are randomly assigned in a 1:1 ratio to receive either FSN treatment (*n* = 22) or pharmacological treatment (*n* = 22) for 4 weeks. Outcome measures are assessed for all participants at baseline, on day 28 after the initiation of intervention, and on days 28, 56, 84, 112, 140 and 168 after treatment completion. Additionally, NIRS measurements are performed for participants in the FSN group during the 1st, 3rd, 5th, and 7th treatment sessions within the therapeutic cycle.

### 2.3 Participants

#### 2.3.1 Inclusion criteria

1. Meeting the diagnostic criteria for migraine without aura (MwoA) according to the International Classification of Headache Disorders, 3rd edition (ICHD-3) (1);
2. Aged 18–65 years;
3. A history of migraine for at least 1 year;
4. At least 2 migraine attacks per month (meet the diagnostic criteria for migraine without aura (criteria B–D) as defined by ICHD-3);
5. No prophylactic medications or other treatments for migraine within the past month;
6. Visual Analog Scale (VAS) score >3;
7. Voluntarily provided written informed consent.

#### 2.3.2 Exclusion criteria

1. Diagnosed with other primary headaches such as neuropathic headache, cluster headache, or tension-type headache;
2. Comorbid secondary headaches or other neurological disorders (e.g., headache due to anxiety, depression, Parkinson’s disease, or intracranial lesions);
3. Uncontrolled or undiagnosed systemic diseases that may interfere with the study, including severe hepatic, renal, cardiovascular, or cerebrovascular diseases; poorly controlled or untreated hypertension (systolic blood pressure exceeding 160 mmHg and/or diastolic blood pressure above 100 mmHg); poorly controlled or untreated diabetes mellitus; malnutrition; hyperthyroidism; or hepatic insufficiency;
4. Comorbid uncontrolled psychiatric disorders;
5. Presence of cervical spine disorders that may affect the assessment of neck and shoulder muscle function.
6. Pregnancy, lactation, or women in the preparation, menstrual, or menopausal phase;
7. Use of prophylactic medications or other treatments for migraine within the past month;
8. History of allergy to the trial medications or contraindications to flunarizine hydrochloride or ibuprofen;
9. Kin lesions at the FSN operation site;
10. Occupations involving driving, working at heights, or other high-risk activities;
11. Refusal to undergo FSN therapy or oral flunarizine hydrochloride.

### 2.4 Sample size calculation

This study is an exploratory investigation, with the primary outcome measure defined as the reduction in the monthly migraine days (MMD) from baseline after 4 weeks of treatment. As a novel acupuncture technique, Fu’s Subcutaneous Needling (FSN) inevitably lacks high-quality randomized controlled trials (RCTs) to inform sample size estimation. Therefore, reference is made to an internationally rigorous RCT on acupuncture for migraine without aura (23), and the sample size is estimated based on this study, combined with clinical FSN practice and the current study design. The hypothesis posited comparable effect sizes between the two interventions (FSN group and flunarizine hydrochloride group) in this trial and those observed in the prior study’s acupuncture and flunarizine hydrochloride groups. The referenced study reported that after 4 weeks of treatment, the MMD in the acupuncture group decreased by 4.1 ± 3.5 from baseline, while the MMD in the flunarizine hydrochloride group decreased by 1.9 ± 2.3 from baseline. To verify whether the efficacy of FSN is at least not inferior to that of flunarizine hydrochloride, a non-inferiority test is employed. Based on previous research (24), the most commonly selected non-inferiority margin for Monthly Migraine Days (MMD) in migraine studies is 1. The calculation is performed using the Non-Inferiority Tests for the Difference Between Two Means in PASS 15.0 software. With *α* = 0.05 (two-sided), power (1−*β*) = 0.9, and an anticipated 25%dropout rate, a total of 44 participants (22 per group, 1:1 ratio) are required to detect a statistically significant difference in MMD changes between the two groups.

### 2.5 Randomization and blinding

All eligible participants who provide informed consent are randomized in a 1:1 ratio to either the FSN group (*n* = 22) or the drug group (*n* = 22). Randomization is performed using a random number table with allocation concealment. The randomization sequence (seed 12345) is generated by methodology personnel not involved in treatment or evaluation using SAS 9.4 software, with a total of 44 random numbers selected. Forty-four allocation cards are prepared, each labeled with a sequential number and a corresponding random number. The cards are sealed in opaque envelopes marked with matching sequential numbers. The envelopes are kept by the statistician. Upon enrollment, the methodologist open the envelope corresponding to the participant’s sequential number and assign the participant to a group based on the random number.

Participant recruitment, intervention administration, outcome assessment, and statistical analysis are conducted independently by different researchers. Due to the nature of acupuncture, neither participants nor practitioners are blinded, but they are instructed not to disclose group allocation during follow-up assessments. To minimize measurement and subjective biases, a unified operational manual is developed to standardize all outcome assessment procedures. Outcome assessors and data collectors are blinded to group allocation and underwent a two-day training program, during which they are instructed not to communicate with participants regarding the intervention. Participants are also directed not to disclose their treatment assignments to the assessors and instructed to record symptoms based on actual experiences, avoiding the influence of treatment expectations. Data analysis is performed by blinded statisticians.

## 3 Intervention

Before formal intervention, researchers record participants’ demographic and clinical characteristics, including gender, age, allergy history, marital and reproductive history, family history, smoking and alcohol consumption history, past medical history, medication history, and other relevant clinical data. Participants are educated on adherence to scheduled FSN sessions or flunarizine hydrochloride administration and instructed to avoid other migraine treatments that might interfere with efficacy assessment, such as oral analgesics, acupuncture, massage and so on. On study days, participants are advised to abstain from caffeine and alcohol and ensure adequate rest the night before.

### 3.1 FSN therapy

Participants are seated while the practitioner identify affected muscles (sternocleidomastoid, scalene, splenius capitis, and upper trapezius) via sliding palpation. The palpation reveal tissue tension, rigidity, firmness, and a smooth, resilient texture, accompanied by cord-like changes. The affected muscle tissues may exhibit localized soreness and discomfort. After disinfection, a disposable FSN needle (diameter 0.60 mm, length 50 mm; Nanjing Paifu Medical Technology Co., Ltd., Jiangsu, China) is inserted parallelly to the subcutaneous loose connective tissue surrounding the affected muscle. Once fully inserted, the soft tube stand is secured in the slot. The operator then perform alternating sweeping motions using the index and ring fingers with an angle of 60°at a frequency of 100 sweeps per minute. This is accompanied by reperfusion activities—resistance-based contraction and relaxation of the affected muscles, performed collaboratively by the practitioner and participant. After 20 sweeping dispersions, a 10-s reperfusion activity is performed, with this procedure repeated 3 times for each affected muscle. Reperfusion methods for specific muscles are as follows:

1. Sternocleidomastoid: seated, the participant turn the head toward the unaffected side while the practitioner apply resistance to the affected side, prompting the participant to attempt turning toward the affected side.
2. Scalene: seated, the participant tilted the head slightly toward the affected side while the practitioner resist, prompting the participant to attempt further tilting.
3. Splenius Capitis: seated, the participant extend the head maximally (until the line between the ear apex and earlobe was horizontal) while the practitioner resist at the occiput, prompting the participant to attempt further extension.
4. Upper Trapezius: seated, the participant maximally elevate the affected shoulder while the practitioner resist, prompting the participant to attempt further elevation.

The FSN group receive treatment twice weekly for 4 weeks.

### 3.2 Oral flunarizine hydrochloride

Participants in the drug group are administered 5 mg flunarizine hydrochloride capsules nightly before sleep for 4 weeks and complete a medication monitoring form after each dose. After the 28th doses, participants will submit their medication monitoring forms to the investigators for review. Close follow-up will be conducted by the investigators during the medication period, and treatment will be immediately discontinued upon occurrence of any adverse events. If necessary, symptomatic treatment will be administered until the adverse events resolve.

Management of Missed Doses: a missed dose is defined as failure to administer the 5 mg dose within a 24-h period. Subjects are required to record each administration in their medication diary. If a dose is missed, no additional dose is to be taken to compensate; instead, the regular dosing schedule is to be resumed at the next planned administration time. All instances of missed doses are recorded in the diary and considered in the adherence analysis.

Study personnel review the medication diaries at each follow-up visit. Adherence is calculated as the percentage of actual doses taken relative to the total planned number of doses (28 doses). Subjects with an adherence rate below 80%were flagged for handling in the sensitivity analysis.

In cases of severe pain (VAS score >8), participants are permitted to take acute analgesic medication. In accordance with the Chinese Guidelines for Migraine Diagnosis and Treatment (25), ibuprofen sustained-release capsules are recommended for pain relief (one capsule per dose, no more than twice daily). If ibuprofen is ineffective, participants could take previously effective analgesics, with detailed records in headache diaries, including drug name, administration time, dosage, and time to headache relief after medication. Prophylactic migraine medications are prohibited.

## 4 Outcomes

### 4.1 Primary outcome

The change from baseline to the end of treatment (Day 28) in monthly migraine days (MMDs).

### 4.2 Secondary outcomes

1. Migraine-Specific Quality of Life (MSQOL) scores, including three dimensions: functional limitations (the degree to which migraines limit daily activities), dysfunction (the avoidance of certain activities due to the concern of migraine attacks), and emotional impact (the effect of migraines on mood), assessed via 14 items on a 6-point scale (0–5). Scores are calculated for each dimension.
2. Headache diaries, recording daily migraine episodes, including presence/absence of headache, frequency, average duration, and use of acute analgesics (if taken, drug name, dosage, and frequency were documented). The reduction rate in rescue medication use was extracted as an independent outcome measure, calculated as the percentage decrease in monthly medication frequency and dosage from baseline to the end of treatment and throughout the follow-up period.
3. Migraine Disability Assessment (MIDAS): This score evaluates migraine-related disability through five items (including the number of days that work, household chores, and social/leisure activities are affected by migraine; the score ranges from 0 to 270, with higher scores indicating greater disability).
4. The difference in Visual Analog Scale (VAS) scores from baseline at week 4 of treatment.

### 4.3 Exploratory measures

#### 4.3.1 Near-infrared spectroscopy (NIRS) imaging

Near-infrared spectroscopy (NIRS) is a technique specifically designed for continuous, real-time monitoring of cortical hemodynamic changes during interventions, making it suitable for therapeutic modalities such as acupuncture or intravenous infusion, which elicit immediate and sustained physiological effects. In contrast, flunarizine hydrochloride is administered orally once daily at bedtime, and its pharmacological effects are cumulative and not temporally aligned with the NIRS measurement period. Consequently, NIRS is unable to effectively capture its underlying mechanisms in real time.

NIRS non-invasively measures real-time changes in cerebral oxygenated hemoglobin (O_2_Hb), deoxygenated hemoglobin (HHb), total hemoglobin (tHb), tissue saturation index (TSI). TSI is defined as (O_2_Hb/tHb) × 100%, quantifying the percentage of tissue oxygenation. It is automatically calculated and displayed in real-time on the PortaLite software interface.

Under normal conditions, increased neuronal metabolic demand elevates regional cerebral blood flow disproportionately to oxygen consumption, resulting in a characteristic NIRS pattern of increased O_2_Hb and decreased HHb concentrations (26). The prefrontal cortex (PFC, excluding primary and secondary motor cortices) plays a key role in pain response and subjective perception modulation, serving as a regulatory region for opioid and other analgesic mechanisms (27). Previous studies (28, 29) demonstrated that during head-down positioning or task execution, migraine patients exhibited significantly smaller increases in tHb and mean O_2_Hb concentrations compared to healthy controls, suggesting potential PFC impairment or diminished vasodilatory responses (29).

This study employ a portable NIRS device (PortaLite MKII) for continuous real-time monitoring of the affected PFC on days 1, 8, 15, and 22 of treatment. Parameters include O_2_Hb, HHb, tHb, and TSI.

##### 4.3.1.1 Procedure

1. Preparation: participants are acclimated for 10 min in a quiet, dimly lit room to stabilize physiological baselines. Caffeine and alcohol intake are prohibited for 24 h prior to measurement, and strenuous exercise is forbidden to minimize systemic hemodynamic fluctuations. The PortaLite NIRS device is calibrated according to the manufacturer’s instructions prior to each use.
2. Test: participants sit upright with the PortaLite secured over the affected PFC, positioned according to the Fp1/Fp2 landmarks of the international 10–20 EEG system (30). Sampling frequency is set at 50 Hz, and optodes are shielded with black cloth to minimize ambient light interference. A head fixation strap is used to control motion artifacts during recording. Drift and noise are eliminated during post-processing using filtering techniques. After a 5-min eyes-closed resting adaptation period to stabilize physiological baselines, a 5-min resting-state recording is obtained. Following FSN needle insertion into the upper trapezius (three needle sets with retention), a 5-min post-treatment resting-state recording is acquired. Additional FSN is administered to other target muscles post-measurement.

### 4.4 Follow-up and recurrence assessment

Outcome measures are assessed at baseline (Day 0), at the end of treatment (Day 28), and during the post-treatment follow-up period (Days 28, 56, 84, 112, 140, and 168) to monitor the long-term durability of efficacy and the recurrence of symptoms.

Recurrence is defined as a return of monthly migraine days (MMD) to ≥80% of the baseline level during the follow-up period, as recorded in headache diaries. In the event of recurrence, management included referral for clinical follow-up (e.g., re-evaluation in a neurology outpatient clinic) and documentation of acute medication use (e.g., ibuprofen), without re-intervention to assess the natural persistence of treatment effects. Recurrence events are analyzed as a secondary endpoint.

## 5 Safety assessment

The primary clinical adverse reactions associated with Fu’s acupuncture needling are subcutaneous hemorrhage and needling syncope. Before the procedure, the operator should avoid needling in areas with dense vascularization. Upon completion of each Fu’s acupuncture session, prolonged pressure should be applied to the needle insertion site and along the needle track to prevent subcutaneous hematoma. Subcutaneous hematomas are graded according to the CTCAE criteria as follows: Grade 1 (Mild): Small hematoma with a diameter <2 cm; managed with local compression or observation. Grade 2 (Moderate): Hematoma measuring 2–5 cm; managed with compression, heat therapy, and temporary discontinuation of treatment if necessary. Grade 3 (Severe): Hematoma >5 cm with persistent bleeding; treatment is discontinued, and the patient is referred for clinical management.

In cases of needling syncope, the needles should be immediately removed. The participant should be placed in a supine position with legs elevated, and vital signs should be monitored. Symptomatic management, such as oxygen administration and hydration, should be provided as needed. Severe cases require prompt medical attention.

Flunarizine’s frequent adverse events are sedation, weight gain and extrapyramidal reactions (31). Body weight is measured every 2 weeks (on days 1, 14, and 28) to quantify weight increase (a change of ≥5% from baseline is considered clinically significant). Extrapyramidal symptoms (EPS), including tremor, akathisia, dystonia, and rigidity, are assessed using the Rating Scale for Extrapyramidal Side Effects (RSESE). Evaluations are conducted at baseline, Week 2, Week 4, and during follow-up by trained neurologists.

Criteria for drug discontinuation included persistent sedation (lasting ≥3 days and interfering with daily activities), moderate to severe EPS (CTCAE grade ≥2), or weight gain of ≥7% accompanied by the patient’s request to withdraw. All events are recorded in patient diaries and reviewed weekly by assessors.

All serious adverse events (SAEs) must be reported to the Ethics Committee within 24 h.

## 6 Statistical analysis

Statistical analyses are conducted using SPSS 25. All tests employ two-sided comparisons, with statistical significance defined as *p* < 0.05.

Normally distributed continuous variables are presented as mean ± standard deviation (SD), whereas non-normally distributed variables are reported as median and inter-quartile range (IQR). Categorical variables appear as frequency and percentage (proportions).

For the MMD, MSQOL, MIDAS and VAS scale scores, between-group differences in changes from baseline to 4 weeks post-treatment are analyzed using either the independent *t*-test or Mann–Whitney U test, as appropriate. Intra-group comparisons between baseline and 4-week post-treatment measurements are conducted using paired *t*-test or Wilcoxon signed-rank test. Given the repeated measurements of MMD and the scale scores, repeated-measures analysis of variance (ANOVA) is additionally employed for supplementary analysis. For near-infrared spectroscopy (NIRS)-derived oxygen saturation levels, analyzed for within-group differences before and after treatment using paired *t*-test.

## 7 Discussion

This study evaluate the efficacy of FSN versus flunarizine hydrochloride in improving pain, reducing headache frequency, and enhancing quality of life in patients with MWoA, while also comparing the adverse event profiles of both interventions. Additionally, the potential mechanisms underlying FSN therapy for migraine without aura are explored.

The treatment of migraine primarily involves preventive and acute-phase therapies. Preventive therapy can decrease the frequency, duration, and severity of migraine attacks, enhance patients’ quality of life, mitigate psychological comorbidities associated with frequent or chronic headaches, decrease reliance on such therapies, and prevent medication-overuse headaches (9). Based on clinical observations, empirical evidence, and supporting literature, commonly used and effective preventive medications for migraine include calcium channel blockers (e.g., flunarizine hydrochloride), antihypertensives (e.g., β-blockers), antiepileptics (e.g., topiramate, carbamazepine), and antidepressants (e.g., amitriptyline, fluoxetine) (1). However, most of these drugs are not specifically developed for migraine and exhibit tolerability and safety concerns, leading to poor patient adherence and challenges in migraine prevention.

Thus, identifying effective preventive treatments with minimal adverse effects is increasingly important. Emerging evidence highlights the role of muscular dysfunction in migraine without aura (9, 10, 32). As a novel acupuncture technique, FSN offers unique advantages. In FSN theory, “affected muscles” are defined as muscles or muscle segments exhibiting pathological functional changes (e.g., abnormal tension despite normal motor control) (33), analogous to myofascial trigger points (MTrPs) (34). Unlike trigger point therapy, FSN addresses functionally impaired muscles or abnormal muscle regions identified via palpation—rather than discrete “points”—through sweeping and reperfusion techniques. This approach significantly expands and accelerates muscle relaxation, improves local blood flow (35), and rapidly relieves functional pathological changes caused by ischemic muscle tension. In previous studies on trigger point therapy for migraine without aura, the sternocleidomastoid, trapezius, and splenius cervicis muscles are commonly selected as target muscles. During ischemic compression of trigger points in these muscles, sympathetic nerve activity in the trigger point region is suppressed, leading to reactive hyperemia (36). This process increases peripheral blood flow and facilitates the clearance of harmful substances within the muscle tissue (37), thereby alleviating pain—a mechanism that aligns with the reperfusion principle of FSN. Consequently, we hypothesize that FSN may be effective in migraine treatment.

The strengths of our study design include: (1) a rigorous comparative evaluation of FSN against the standard preventive drug flunarizine hydrochloride to assess its efficacy in migraine without aura, providing evidence for complementary and alternative therapies; and (2) the first application of near-infrared spectroscopy (NIRS) to measure cerebral cortical hemodynamic parameters, exploring FSN’s mechanism of action from a microcirculatory perspective, which may offer insights for further research and clinical application.

This study has several limitations. This study is a single-center trial with a small sample size (*n* = 60), which may be subject to selection bias, thereby limiting the generalizability of its findings to different populations or settings. Due to the inherent characteristics of acupuncture and pharmacotherapy, blinding both practitioners and participants presents considerable challenges, potentially increasing the risk of bias. To mitigate this risk, the intervention operators, outcome assessors, data collectors, and statisticians are all independent researchers. All personnel, except the operators, remained blind to group allocation. Standardized operating procedures are implemented to ensure adherence to treatment and assessment protocols, thereby minimizing unintended influences on outcome measures. The use of rescue medications may interfere with the evaluation of headache intensity and frequency; therefore, the final analysis will comprehensively document and compare intergroup medication usage (dosage, frequency, time to onset). Subsequent studies should establish unified guidelines. Limited by current equipment, near-infrared spectroscopy monitoring focused solely on hemodynamic changes in the prefrontal cortex and did not assess the effects of FSN on other brain regions. As an exploratory study, this trial aimed to investigate whether FSN may reduce patients’ reliance on flunarizine, decrease drug-related side effects, and provide a safe alternative for patients with contraindications to medication. Future large-scale, multi-center clinical trials are warranted to validate the long-term efficacy of FSN and its potential as an adjuvant or first-line treatment.

## 8 Conclusion

The hypothesis of this study is that FSN therapy is not inferior to flunarizine hydrochloride in improving headache intensity and attack frequency in patients with migraine without aura, and that its therapeutic effects may be sustained over time. The study aims to test this hypothesis in a controlled clinical trial. The results may demonstrate the clinical utility of FSN therapy for migraine without aura and facilitate its wider implementation.
